# A Novel Cosmetic Formulation That Rapidly Reduces Hair Shedding in Females

**DOI:** 10.1111/jocd.16592

**Published:** 2024-09-26

**Authors:** Nina Mehta, Daniel do Nascimento Fonseca, Rachita Dhura, Carlos Wambier, Torello Lotti, Andy Goren

**Affiliations:** ^1^ University of North Carolina School of Medicine Chapel Hill North Carolina USA; ^2^ College of Medicine Nilton Lins University Manaus Amazonas Brazil; ^3^ Department of Dermatology LTM Medical College and Hospital Sion Mumbai India; ^4^ Department of Dermatology Alpert Medical School of Brown University Providence Rhode Island USA; ^5^ University of Rome (“G. Marconi”) Rome Italy

**Keywords:** alpha 1 agonist, hair loss, hair shedding, TAAR receptor agonist, traction alopecia, treatment

To the Editor

Hair shedding is common in women, and while the main objective of hair shedding treatment is to address the underlying causes, topical preparations are still needed to prevent hair loss. Non‐autoimmune dermatologic conditions characterized by hair shedding include anagen effluvium, telogen effluvium, traction alopecia (TA), and female pattern hair loss (FPHL) [1]. FPHL and female androgenetic alopecia (FAGA) are forms of female alopecia without and with androgen increases, respectively [2], and FPHL is the most common type of alopecia seen in women, affecting up to one‐third of White adult women [3]. The detrimental psychological impact of hair shedding and hair loss on women's well‐being and quality of life has driven the need for innovative therapies to combat this problem [4].

Alpha‐1 adrenergic receptors play a significant role in regulating smooth muscle tone in blood vessels. Targeting and modifying the alpha‐1 receptor by its agonists can lead to an increased threshold of force required to pull out hair. Current synephrine and tyramine formulations are limited by the prolonged time to onset of therapeutic effect. In addition, these formulations need to be administered at high concentrations and take a relatively long time to achieve the desired results; indeed, our previous study showed that it took an average of 30 min to observe a response [5]. A rapid therapeutic effect is desirable so that hair shedding is reduced while showering or immediately prior to commencing hair treatments.

We assessed the efficacy of combination alpha‐1 adrenergic receptor agonist and trace amine‐associated receptor (TAAR) agonist (DA‐OTC‐002) as a topical cosmetic hair treatment to prevent hair loss. Seventy‐six healthy female subjects were included in the study, and DA‐OTC‐002 or placebo control (vehicle alone) was applied to each side of the scalp before standardized brushing to quantify immediate hair shedding (Figure 1). All subjects provided informed consent during study enrollment. Patients were randomized at a 1:1 ratio using Sealed Envelope software. Statistical analysis was performed using MedCalc v23.0.1.

**FIGURE 1:**
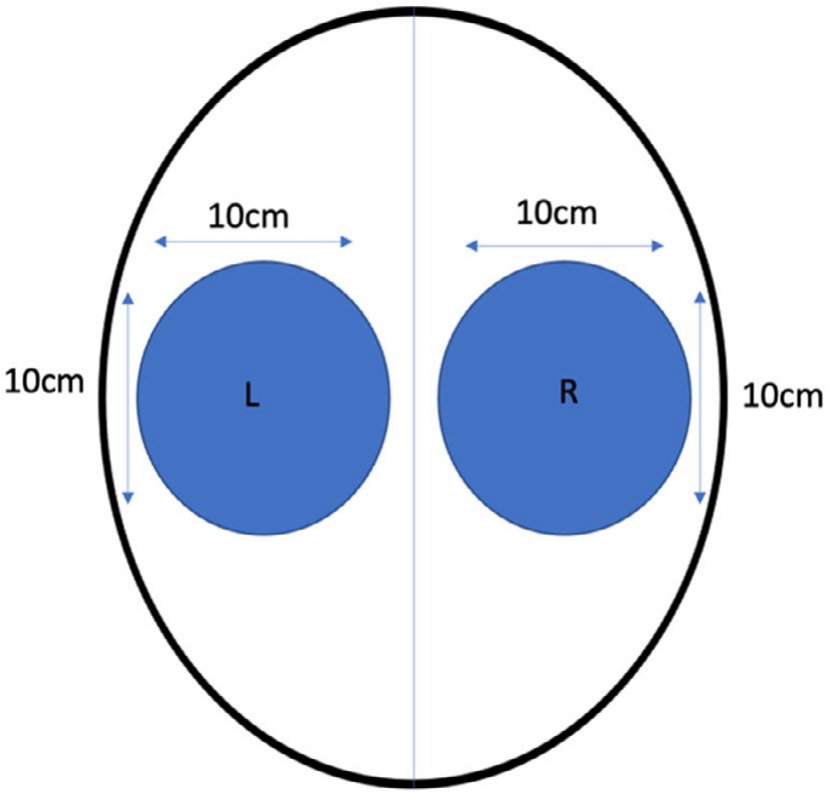
Placement of the formulations on the top of the scalp by the investigator.

In the experimental group, the number of hairs shed was 13.62 ± 11.28, significantly lower than the 36.26 ± 12.74 observed in the placebo group, with a mean difference of 22.63 (*p* < 0.0001) (Table 1). Average hair shedding in the DA‐OTC‐002‐treated area was 62.5% lower than in the placebo‐treated area. No adverse events, such as skin irritation or allergic reactions, were observed.

**TABLE 1:** Number of hairs shed in the treatment and control groups.

	Treatment formula	Placebo formula
Number patients	76	76
Average number hair shed	13.62	36.26

The application of DA‐OTC‐002, a combined selective alpha‐1 agonist (synephrine) and a selective TAAR agonist (tyramine hydrochloride), significantly reduced hair loss after only 5 min of application to the scalp. To our best knowledge, this is the first study to demonstrate the potential beneficial effect of combining an alpha‐1 agonist and a TAAR receptor agonist for the treatment of hair shedding. In future studies, it would be valuable to compare the novel formula with 5% minoxidil, currently the most accepted and recommended treatment for hair loss, which can sometimes lead to an initial increase in shedding followed by a notable reduction in hair loss. While our study did not address long‐term use, it is possible that extended use could treat chronic hair loss. There is no reason to believe that this formulation would cause adverse or rebound effects even with use beyond 12 months, but this needs clinical confirmation. Finally, as the mechanism of action that causes contraction of the arrector pili muscle is similar in both males and females, this formulation should prevent hair loss in males.

## Author Contributions

All listed authors made a significant scientific contribution to the research in the manuscript, approved its claims, and agreed to be an author. D.N.F., R.D., C.W., T.L. performed the research and carried out the experiments. N.M. took the lead in writing the manuscript. All authors contributed to the final version of the manuscript. A.G. conceived the original idea and supervised the project.

## Conflicts of Interest

The authors declare no conflicts of interest.

## Data Availability

The data that support the findings of this study are available from the corresponding author upon reasonable request.
